# miR-615-3p promotes the epithelial-mesenchymal transition and metastasis of breast cancer by targeting PICK1/TGFBRI axis

**DOI:** 10.1186/s13046-020-01571-5

**Published:** 2020-04-26

**Authors:** Bo Lei, Dandan Wang, Ming Zhang, Yuwei Deng, Huijie Jiang, Yiwen Li

**Affiliations:** 1 Department of Breast Surgery, Harbin Medical University Cancer Hospital, 150 Haping Road, Harbin, 150086 China; 2 Department of Radiology, The Second Affiliated Hospital of Harbin Medical University, 246 Xuefu Road, Harbin, 150086 China; 3 Department of Oncology, The Second Affiliated Hospital of Harbin Medical University, 246 Xuefu Road, Harbin, 150086 China

**Keywords:** miR-615-3p, EMT, TGF-β, PICK1, Breast cancer

## Abstract

**Background:**

Increasing evidence indicates that epithelial-mesenchymal transition (EMT) can be regulated by microRNAs (miRNAs). miR-615-3p was shown to be involved in tumor development. However, the role of miR-615-3p in the metastasis of breast cancer remains largely unknown.

**Methods:**

The expression of miR-615-3p in breast cancer cells and tissues was assessed by qRT-PCR and situ hybridization assays. Effects of miR-615-3p on tumor metastasis were evaluated with experiments in vitro and mouse model. EMT markers were detected by western blot and immunofluorescence assays. Molecular mechanism of miR-615-3p in the regulation of breast cancer cell metastasis was analyzed by Western Blot, Co-immunoprecipitation, and Luciferase assay.

**Results:**

In the present study, we found that miR-615-3p was significantly elevated in breast cancer cells and tissues, especially in those with metastasis. In breast cancer cell lines, stable overexpression of miR-615-3p was sufficient to promote cell motility in vitro, and pulmonary metastasis in vivo, accompanied by the reduced expression of epithelial markers and the increased levels of mesenchymal markers. Further studies revealed that the reintroduction of miR-615-3p increased the downstream signaling of TGF-β, the type I receptor (TGFBRI) by targeting the 3′-untranslated regions (3′-UTR) of PICK1. PICK1 inhibits the binding of DICER1 to Smad2/3 and the processing of pre-miR-615-3p to mature miR-615-3p in breast cancer cells, thus exerting a negative feedback loop.

**Conclusions:**

Our data highlight an important role of miR-615-3p in the molecular etiology of breast cancer, and implicate the potential application of miR-615-3p in cancer therapy.

## Background

Breast cancer is the most commonly diagnosed cancer, it accounts for 11.6% of the total cases and 6.6% of all cancer death in the world [1]. Although surgical removal of the tumor is still the primary treatment of choice, apart from radiotherapy or surgery, chemotherapy remains the most effective way for preventing cancer cell growth and metastasis thereby enhancing the survival of cancer patients [2]. Some evidence showed that natural products may play a potential and promising role in the development of novel chemotherapeutics for the treatment of cancers [3]. The transforming growth factor-β (TGF-β) family of cytokines regulates many processes such as immune suppression, angiogenesis, wound healing, and epithelial-mesenchymal transition (EMT) [4]. Upon binding TGF-β, the type I receptor (TGFBRI) binds TGFBRII, which results in the activation of the transcription factors SMAD2 and SMAD3 [5]. In the early stage of tumorigenesis, the proliferation of epithelial cells retains sophisticated sensitivity to TGF-β, wherein TGF-β elicits a tumor-suppressive response [6]. However, transformed cells become refractory to TGF-β mediated growth inhibition and acquire a phenotype wherein the intracellular signaling circuitry is altered, leading to tumorigenic and metastatic effects in response to TGF-β [7]. Ligand-dependent activation of TGF-β receptors and regulation of their subsequent kinase activity is a complex process that can involve several posttranslational modifications of the receptors [8]. With so much complexity in the pathway, we set out to find potential new regulators of TGF-β receptor activity.

Invasion and metastasis, two of the most important hallmarks of malignant tumors, are the foremost fatal factors for breast cancer [9]. Identification of invasive and/or metastatic factors and an understanding of the underlying molecular mechanisms may provide novel targets for cancer therapy. Increasing evidence indicates that epithelial-mesenchymal transition (EMT) is a key event in tumor invasion and metastasis [10]. During EMT, a morphological change from epithelial-like to mesenchymal-like appearance is accompanied by loss of cell-cell adhesion and activation of mesenchymal markers, such as N-cadherin, fibronectin and vimentin, as well as increased motility of tumor cells, which consequently facilitates tumor metastasis [11]. Previous mechanistic investigations had demonstrated many regulators that could enhance the EMT state such as c-Fos overexpression [12].

Deregulation of miRNA has been observed in various diseases, including cancer [13]. For instance, previous research has demonstrated that anti-miR-203 suppresses ER-positive breast cancer growth and stemness by targeting SOCS3 [14]. Furthermore, more and more reports have indicated that a few miRNAs suppress (for example, the miR-200 family) or promote (for example, miR-24) EMT and tumor metastasis to date. Although some miRNAs (for example, miR-214) have been identified to regulate EMT in breast cancer, the role of miRNAs in the EMT of breast cancer deserved further investigation [15–17].

Protein interacting with C kinase 1 (PICK1) is a domain-containing protein that inhibits actin-related protein 2/3 (Arp2/3)-dependent actin polymerization, and participates in the regulation of the trafficking of several cell-surface receptors [18]. It was reported that PICK1 negatively or positively regulated the neoplastic infiltration of astrocytic or breast tumors [19, 20]. Especially, it was also suggested that PICK1 may participate in breast cancer development by targeting TGF-β type I receptor (TβRI) for degradation. Moreover, a negative correlation between PICK1 expression and TβRI or p-Smad2 levels is observed in human breast tumors [21]. However, very little was known about the relationship between miRNA and PICK1.

It was reported that miR-615-3p may function as a potential tumor suppressor through diverse mechanisms [22, 23]. However, the role of miR-615-3p in the metastasis of breast cancer remains largely unknown. In the present study, we showed that miR-615-3p displayed a more pronounced increase in breast cancer tissues and cells. The knockdown of miR-615-3p expression significantly repressed the in vitro migration and invasion, and in vivo pulmonary metastasis of breast cancer cells. Subsequent mechanism studies revealed that miR-615-3p promoted EMT program by directly targeting PICK1, and consequently increased the accumulation of Vimentin and decrease of E-cadherin, which promoted EMT. These findings provided novel mechanistic insights into the role of miR-615-3p in EMT and metastasis.

## Materials and methods

### Cell culture

The human breast cancer cell lines MCF-7, BT-549, T47D, MDA-MB-468, MDA-MB-231, and HEK293T were purchased from American type culture collection (ATCC, USA). Cell lines were authenticated on the basis of viability, recovery, growth, and morphology. The expression status of ER was further confirmed by western blotting before they were used in the experiments. All cells were cultured in Dulbecco’s modified Eagle’s media medium containing 10% fetal bovine serum (Hyclone) at 37 °C with 5% CO_2_ in cell culture incubators.

### Plasmids

Cultured cells were transfected with miRCURY LNA Inhibitor Control (Negative control A catalog#199004–00, 5 nmol, EXIQON), miRCURY LNA Inhibitor (hsa-miR-615-3p catalog#411356–00, Batch#224134, 5 nmol, EXIQON), mirVana microRNA mimic negative control #1(catalog#4464058, Lot#ASO0VDIQ, 5 nmol, Ambion), mirVana microRNA mimic hsa-miR-615 (catalog#4464066, 5 nmol, Ambion). Using Ambion by Life Technologies Silencer siRNA Labeling Kit-Cy3 (cat # AM1632) according to manufacturer’s instructions. Expression plasmids of miR-615-3p were created by PCR amplification using human genomic DNA as a template. The primers are as follows: miR-615-3p, forward 5′ CCC AAG CTT GGG CAT AAT TGG ATC ATA GGA AC 3′ and reverse 5′ CCG GAA TTC CGG GTG AAT AGC TTG CAG CGT TC 3′. The reactions were incubated in tubes with 30 cycles of 98 °C for 10 s and 64 °C for 30 s. The PCR product (518 bp) was digested with *Hin*dIII and *Eco*RI, followed by insertion into a *Hin*dIII- and *Eco*RI-open pcDNA 3.1(+) vector (Invitrogen) and confirmed by DNA sequencing.

### Luciferase assay

The region of the wild-type *PICK1* mRNA 3′UTR with a putative miR-615-3p binding site or mutant *PICK1* mRNA 3′UTR were cloned into pMIR-REPORT Luciferase vector (cat # AM5795, Applied Biosystems) using *Spe I* and *Hind III* sites. The sequences of the putative binding site and the regions targeted by mutagenesis and cloned into the reporter gene. All plasmids were verified by sequencing. These constructs were transfected into indicated cells using Lipofectamine LTX with Plus Reagent (cat #18324–012, Life Technologies). Cells were plated at a density of 3600/cm^2^ {(1 × 10^4^) per well, into a 96-well plate and attached overnight. They were co-transfected with 100 ng of wild-type or mutant reporter vector, 10 ng of internal control pRL-TK-Renilla-luciferase plasmid (cat# E2241, Promega) and negative control #1 or mirvana microRNA miR-615-3p mimic, both from Life Technologies final concentration, 80 nM. Twenty- four hours post-transfection, luciferase activities were measured using the Dual-Glo Luciferase Assay System (cat # E2920, Promega) according to the manufacturer’s instructions. Firefly luciferase values were normalized by dividing by the *Renilla* luciferase values.

### Quantitative real-time PCR (qRT-PCR)

Total RNA was isolated with Trizol reagent (Invitrogen, USA), according to the manufacturer’s instructions. Total RNA from each sample was reverse transcribed with oligo (dT)_20_ using SuperScript III Reverse Transcriptase (Invitrogen, USA) followed by real-time PCR. Real-time PCR was performed with SYBR Green PCR Master Mix reagents using an ABI Prism 7700 Sequence Detection System (Applied Biosystems, USA). Data were analyzed according to the comparative Ct method. U6 was used as an internal reference for miRNAs and β-actin as used as an internal reference for mRNAs. The primers are as follows: miR-615-3p, forward: 5′-ACA CTC CAG CTG GGT CCG AGC CTG GGT CTC-3′, reverse: 5′-TGG TGT CGT GGA GTC G-3′; PICK1 mRNA, forward 5′-TAC TAA CAG CGA GCT TCC GC-3′ and reverse 5′-GGT TCC GAG AGT TGG AGT GG-3′; β-actin mRNA, forward 5′-AGA GAT GGC CAC GGC TGC TT-3′ and reverse 5′-ATT TGC GGT GGA CGA TGG AG-3′; U6, forward 5′-CTC GCT TCG GCA GCA CA-3′ and reverse 5′-AAC GCT TCA CGA ATT TGC GT-3′.

### Co-immunoprecipitation, western blot assay, and antibodies

Co-immunoprecipitation assays were carried out by using the Pierce Co-Immunoprecipitation Kit (#26149, Thermo Fisher, USA) according to the manufacturer’s protocol. Western blotting was performed according to the previously described procedures [24]. The cells were lysed in lysis buffer. Protein was separated by SDS-PAGE (10% gels) and transferred onto a 0.22 μm polyvinylidene fluoride (PVDF) membrane. The proteins were probed with specific antibodies overnight. After incubation, the blots were incubated with corresponding anti-rabbit IgG H&L (HRP) or anti-mouse IgG H&L (HRP) for 1 h at room temperature. The proteins were detected using ECL western blot detection system. Anti-PICK1(#ab3420,rabbit) antibody, anti-E-cadherin(#ab15148, rabbit) antibody and anti-vimentin (#ab16700, rabbit) antibody were obtained from Abcam. Anti-smad2(#5339 rabbit) antibody, anti-p-smad2(#3108 rabbit ser465/467) antibody, anti-p-smad3(#9520 rabbit ser423/425) antibody, anti-smad3(#9523 rabbit) antibody, and anti-Dicer (#5362 rabbit) antibody were purchased from Cell Signaling Technologies (CST). Anti-TGFβ RI (#sc-101,574, mouse) antibody and anti-TGFβ RII (#sc-17,791, mouse) antibody were obtained from Santa Cruz Biotechnology. Anti-β-actin (#WL01372, mouse) was obtained from Wanleibio (China).

### Human tissue analysis

Breast tumor and adjacent noncancerous tissues were obtained from the Affiliated Hospital of Harbin Medical University, with the informed consent of patients and with approval for experiments from the Affiliated Hospital of University.

### Animal experiments

Indicated cells (2 × 10^6^) that stably expressed miR-615-3p or control plasmids were injected into the tail vein of 6-week-old female nude mice. All mice were sacrificed 8 weeks after the injection. All the experimental procedures involving animals were conducted in accordance with Institutional Animal Care guidelines and approved ethically by the Administration Committee of Experimental Animals.

### Cell migration and in vitro invasion

The migration and in vitro invasion were performed in 24-well cell culture inserts (Corning Life Sciences). The inserts for invasion assays were coated with 30 μL of Matrigel matrix at 37 °C for 1 h. Cells (1 × 10^5^ cells per Transwell) were added into the upper chamber and allowed 24 h for cell migration. For in vitro invasion, 2 × 10^5^ cells were added into each upper chamber and allowed 24 h for invasion. After the period of migration or invasion, cells on the undersurface of the upper units were stained and counted under a phase-contrast microscope.

### Immunofluorescence staining

Cells were cultured on coverslips overnight and fixed with 4% paraformaldehyde, followed by the treatment of 1% Triton X-100 (Thermo Fisher, MA) for permeabilization. To visualize the E-cadherin and vimentin, we incubated the coverslips with the respective antibodies for 1 h and then rhodamine-conjugated secondary antibody for another hour. The fluorescence staining was observed with the aid of a fluorescence microscope (Axiovert 200 M; Carl Zeiss, USA). 4′,6-diamidino-2-phenylindole (DAPI) was included during staining to visualize nuclei of cells.

### Statistical analysis

Data were presented as the means ±S.E.M. from three or more independent experiments unless indicated otherwise. Statistical analysis was performed using two-tailed t-tests. *P* < 0.05 was considered a significant difference.(*P < 0.05; **P < 0.01; ***P < 0.001).

## Results

### The miR-615-3p level is upregulated in breast cancer tissues and breast cancer cell lines, and positively correlated with the metastatic ability of breast cancer cells

**Fig. 1:**
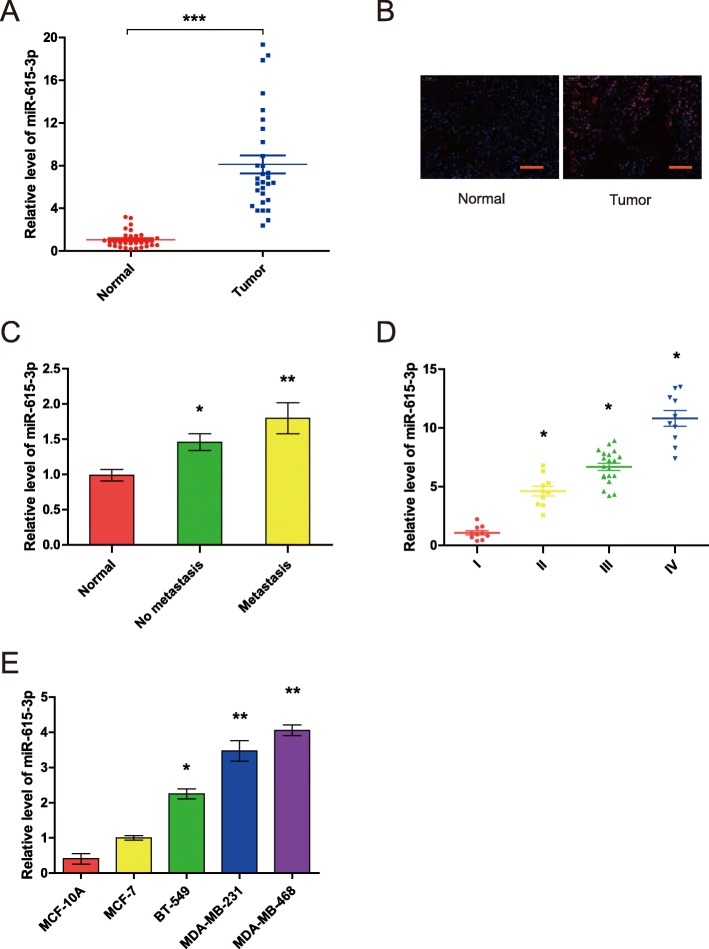
The miR-615-3p level is upregulated in breast cancer tissues and breast cancer cell lines and positively correlated with the metastatic ability of breast cancer cells. (**a**) qRT-PCR analysis of miR-615-3p expression in human breast cancer tissue samples and their matched normal breast tissues from 30 breast cancer patients. (**b**) In situ hybridization analysis of miR-615-3p expression in human breast cancer tissues and matched normal tissues (scale bars = 100 μm). (**c**) Relative expression of miR-615-3p in metastases and non-metastases tissues(N (Normal, No metastasis, Metastasis) = 51,31,20 respectively). (**d**) Relative expression of miR-615-3p with the progression of the clinical stage of breast cancer(N(I, II, III, IV) = 11,10,20,10 respectively). (**e**) qRT-PCR analysis of miR-615-3p expression in noncancerous human mammary epithelial cells (MCF-10A) and breast cancer cell lines (MCF-7, BT-549, MDA-MB-231, and MDA-MB-468 cells) with different metastatic potential

To investigate the role of miR-615-3p in breast cancer pathogenesis, we determined the expression of miR-615-3p in breast cancer tissues and adjacent normal tissues by using qRT-PCR. We observed that miR-615-3p expression was significantly increased in breast cancer tissues compared with adjacent normal breast tissues (Fig. 1a). In situ hybridization assays indicated that miR-615-3p was highly expressed in tumor tissues, whereas little signal was observed in the normal breast tissues (Fig. 1b). We further determined the correlation between the miR-615-3p level and the metastatic status of patients with breast cancer. We found that the expression of miR-615-3p was positively associated with lymph node metastasis (Fig. 1c), and tumors expressed increasing levels of miR-615-3p with the progression of the clinical stage (Fig. 1d) in breast cancer patients. We also used qRT-PCR to measure the expression levels of miR-615-3p in breast cancer cell lines. BT-549, MDA-MB-468, MCF-7, MDA-MB-231 and a non-malignant breast epithelial cell MCF-10A were used. Compared to low metastatic MCF-7 cells, the expression of miR-615-3p was obviously increased in highly metastatic cells, while the expression of miR-615-3p was significantly decreased in non-malignant breast epithelial cell MCF-10A (Fig. 1e). These results suggest that the miR-615-3p level is upregulated in breast cancer tissues and breast cancer cell lines and that it is positively correlated with the metastatic ability of breast cancer cells.

### miR-615-3p promotes breast cancer metastasis

**Fig. 2:**
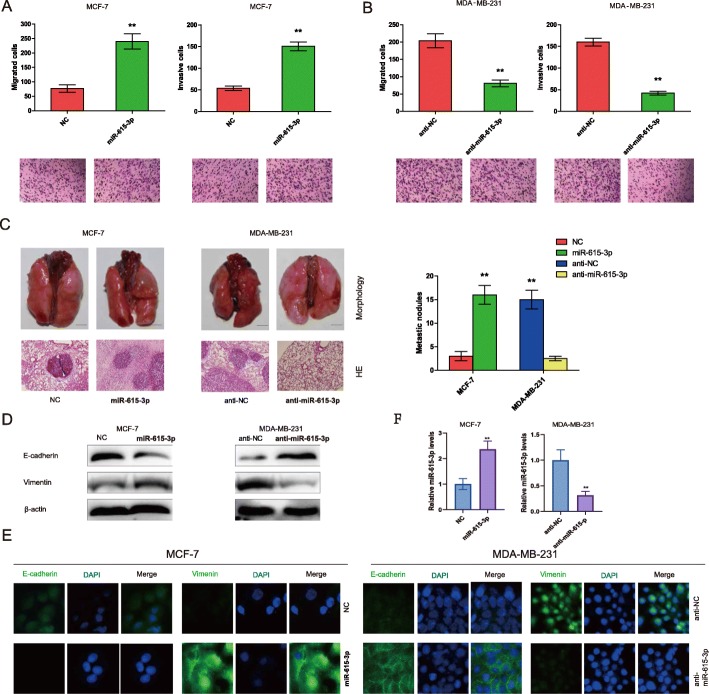
miR-615-3p promotes breast cancer metastasis via EMT. **a** Transwell assay measuring MCF-7 cell migration (left panel) and invasion (right panel) with a control vector (NC) or with ectopic miR-615-3p expression. **b** Transwell assay measuring MDA-MB-231 cell migration (left panel) and invasion (right panel) with a control vector (Anti-NC) or with anti-miR-615-3p expression. **c** Representative images (left upper panel), H&E staining (left down panel), and the number of metastatic nodules (right panel) of lung metastasis. Ectopic miR-615-3p expression promoted lung metastasis of MCF-7 cells (left panel). Knockdown of endogenous miR-615-3p by antisense RNA suppressed lung metastasis of MDA-MB-231 cells (right panel). Western blot (**d** left panel) and immunofluorescence (**e** left panel)) of MCF-7 cells with a control vector (NC) or with ectopic miR-615-3p expression. Ectopic miR-615-3p down-regulated the epithelial marker E-cadherin, up-regulated the mesenchymal marker Vimentin. Western blot (**d** right panel) and immunofluorescence (**e** right panel) of MDA-MB-231 cells with a control vector (Anti-NC) or with antisense RNA against endogenous miR-615-3p (Anti-miR-615-3p). miR-615-3p knockdown suppressed induction of EMT, as indicated by downregulation of Vimentin and upregulation of E-cadherin. **f** The transfection efficiency of MCF-7(miR-615-p overexpression) and MDA-MB-231(anti-miR-615-p) was shown in the image

To evaluate the effect of miR-615-3p on MCF-7 cells, which have low endogenous miR-615-3p expression, we ectopically expressed miR-615-3p in MCF-7 cells. Ectopic miR-615-3p did not promote MCF-7 cell growth (data not shown). However, the Transwell migration assay and Matrigel invasion assay showed that ectopic expression of miR-615-3p increased migration and invasion of MCF-7 cells significantly (Fig. 2a). To investigate whether silencing of miR-615-3p expression in metastatic cells impedes their ability to metastasize, we knocked down endogenous miR-615-3p levels in MDA-MB-231 cells. MDA-MB-231 cells were infected with lentiviral vectors to establish two stable lines that either expressed antisense RNA against miR-615-3p (Anti-miR-615-3p) or contained a control vector (Anti-NC). Transwell migration assay and Matrigel invasion assay showed that knockdown of miR-615-3p decreased migration and invasion of MDA-MB-231 cells significantly (Fig. 2b). The transfection efficiency of MCF-7(miR-615-p overexpression) and MDA-MB-231(anti-miR-615-p) was shown in Fig. 2f.

To investigate whether miR-615-3p promotes metastasis in vivo, we performed intravenous injection through the tail vein with the same cells. In the group injected with MCF-7-miR-615-3p cells, all 10 mice developed lung metastasis, with a large number of metastatic nodules covering the entire lung. In contrast, only 1 out of 10 mice injected with MCF-7-NC cells developed lung metastasis, with a small number of metastatic nodules (Fig. 2c). Therefore, miR-615-3p expression promoted the colonization of circulating MCF-7 cells in the lung. Similarly, equal numbers of MDA-MB-231-Anti-NC and MDA-MB-231-Anti-miR-615-3p were intravenously injected through the tail vein. In the control group (MDA-MB-231-Anti-NC), all 10 mice (10/10) developed lung metastasis, which is consistent with the high metastatic potential of MDA-MB-231. miR-615-3p knockdown significantly reduced lung metastasis of MDA-MB-231 cells. In the MDA-MB-231-Anti-miR-615-3p group, only 2 out of 10 (2/10) mice developed lung metastasis (Fig. 2c). Thus, miR-615-3p knockdown suppressed metastasis of MDA-MB-231 cells in vivo*.*

As an EMT-like process has been associated with breast cancer metastasis [25], we examined whether miR-615-3p promotes EMT. We measured the levels of the epithelial marker E-cadherin and the mesenchymal marker Vimentin in MCF-7-miR-615-3p and MCF-7-NC cells using both western blot analysis and immunofluorescence. Compared with the control of NC cells, which had high E-cadherin expression and low Vimentin expression, MCF-7-miR-615-3p cells had significantly downregulated E-cadherin expression and upregulated Vimentin expression (Fig. 2d and e, left panel). Consistent with the EMT markers, SW480-miR-615-3p cells adopted a spindle-shaped, mesenchymal-like morphology in contrast to the epithelial-like morphology of SW480-NC cells (data not shown). Hence ectopic miR-615-3p expression causes MCF-7 cells to undergo EMT. We then examined whether the silencing of miR-615-3p could impede the ability of cells to undergo EMT. miR-615-3p knockdown reduced Vimentin expression and upregulated E-cadherin expression in MDA-MB-231 cells, as shown by immunofluorescence and western blot (Fig. 2d and e, right panel). Therefore, miR-615-3p knockdown impedes the ability of MDA-MB-231 cells to undergo an EMT-like process.

Collectively, the in vitro migration and invasion assays and in vivo metastasis assays, with ectopic expression of miR-615-3p in MCF-7 cells and knockdown of endogenous miR-615-3p in MDA-MB-231 cells, indicate that miR-615-3p promotes breast cancer metastasis.

### miR-615-3p promotes TGF-β1-induced breast cancer cell migration via enhancing Smad2 and Smad3 activation

**Fig. 3:**
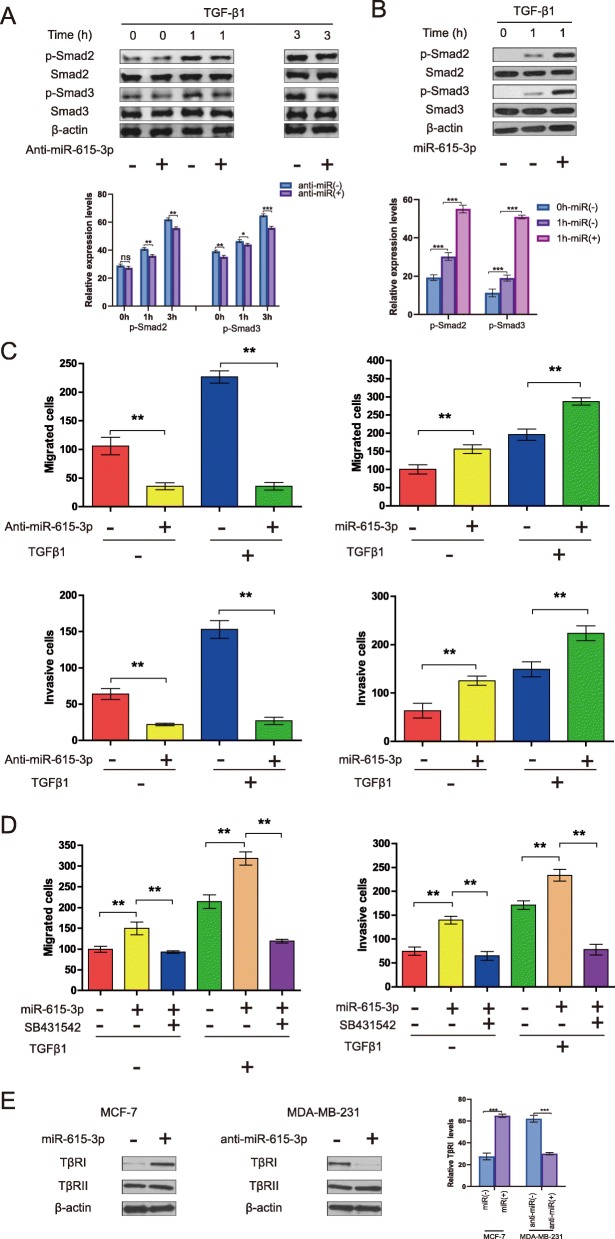
miR-615-3p promotes TGF-β1-induced breast cancer cell migration via enhancing Smad2 and Smad3 activation. **a** MDA-MB-231 cells were transfected with nonspecific control siRNA (Anti-NC) or miR-615-3p-targeted siRNA (Anti-miR-615-3p) and then treated with TGF-β1 (5 ng/ml) for the indicated periods of time. Whole-cell lysates were blotted with the indicated antibodies. Relative protein level listed in lower panel histogram of **(a)** was calculated and compared (Student’s t-test). **b** MCF-7 cells transfected with the control plasmid (NC) or miR-615-3p expression plasmid were treated with TGF-β1 (5 ng/ml) for 1 h. Whole-cell lysates were blotted with the indicated antibodies. Relative protein level listed in lower panel histogram of (**b**) was calculated and compared (Student’s t-test). **c** MDA-MB-231(left panel) cells were transfected with nonspecific control siRNA (Anti-NC) or miR-615-3p-targeted siRNA (Anti-miR-615-3p), MCF-7 cells (right panel) were transfected with control plasmid (NC) or miR-615-3p expression plasmid, underwent Transwell migration and invasion assays in the presence or absence of TGF-β1 (5 ng/ml). **d** MCF-7 cells transfected with the indicated reagents underwent Transwell migration and invasion assays in the presence or absence of SB431542 (10 μM). Columns show the mean of three independent experiments performed in triplicate. **e** MCF-7and MDA-MB-231 cells were transfected with indicated reagents, and whole-cell lysates were then blotted with the indicated antibodies. Relative protein level listed in right panel histogram of Fig. 3**e** was calculated and compared (Student’s t-test)

To determine the molecular mechanism of miR-615-3p in the regulation of breast cancer cell migration, we analyzed the effect of miR-615-3p on the TGF-β1 signal pathway which is a critical EMT inducer in a number of cancer cells including breast cancer cells [26, 27].TGF-β1 treatment induced phosphorylation of Smad2 and Smad3 in a time-dependent manner [28, 29]. However, knockdown of miR-615-3p suppressed this phosphorylation (Fig. 3a). In contrast, overexpression of miR-615-3p resulted in a significant increase in TGF-β1-induced phosphorylation of Smad2 and Smad3 (Fig. 3b), suggesting that miR-615-3p could function as a positive regulator of Smad2 and Smad3 activation in response to TGF-β1.

Although TGF-β1 treatment significantly increased the migratory and invasive potentials of MDA-MB-231 cells, however, knockdown of miR-615-3p inhibited TGF-β1-induced cell migration (Fig. 3c). In contrast, overexpression of miR-615-3p resulted in a significant increase in TGF-β1-induced cell migration and invasion (Fig. 3c), suggesting that miR-615-3p could be involved in the regulation of TGF-β1-mediated responses.

We next investigated whether SB431542, a specific TβRI inhibitor, modulates miR-615-3p-induced cell migration and invasion. Treatment of MCF-7 cells with SB431542 significantly suppressed both basal and miR-615-3p-induced cell migration and invasion of MCF-7 cells (Fig. 3d), suggesting that miR-615-3p could be involved in the regulation of TβRI-mediated responses.

The TGF-β receptor internalization effectively decreasing TGF-β1 signaling [30, 31], we next investigated whether miR-615-3p regulates plasma-membrane expression of TβRI. Western blot analysis revealed that miR-615-3p knockdown in MDA-MB-231 by siRNA decreased the expression level of TβRI, but not that of TβRII (Fig. 3e). In contrast, ectopic expression of miR-615-3p in MCF-7 cells increased the expression level of TβRI, but not that of TβRII (Fig. 3e). However, the TβRI mRNA level remained unchanged (data not shown), suggesting that miR-615-3p may modulate TβRI stability.

### miR-615-3p directly targets PICK1

**Fig. 4:**
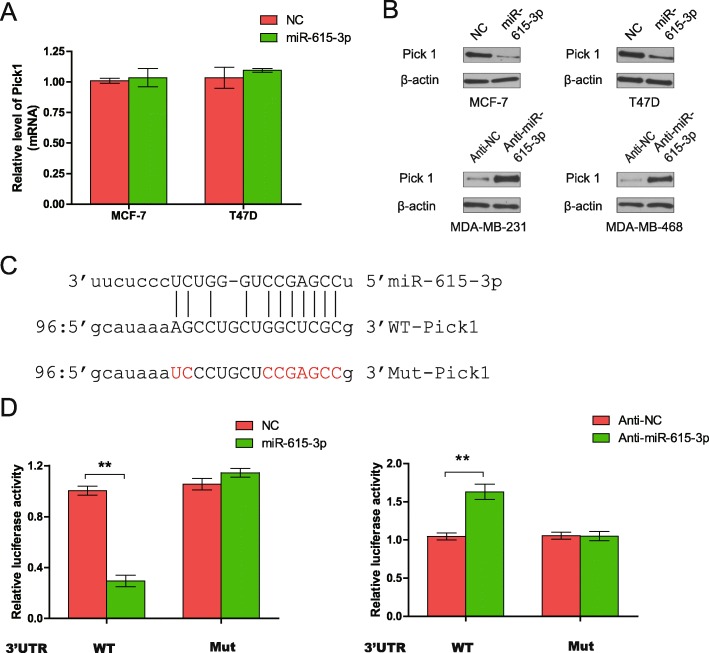
PICK1 is a direct target of miR-615-3p. **a** The real-time PCR analysis of PICK1 mRNA level in MCF-7 and T47D cells infected with miR-615-3p-expressing or control vector. **b** Western blot analysis of PICK1 protein levels in MCF-7 and T47D cells infected with miR-615-3p-expressing or control vector (upper panel). And Western blot analysis of PICK1 protein level in MDA-MB-468 and MDA-MB-231 cells transfected with anti-miR-615-3p or control oligo (lower panel). **c** Gene structure of PICK1 showing the predicted target site of miR-615-3p in its 3′-UTR. **d** MCF-7 cells were transfected with a reporter gene containing wild-type (3′UTR-WT) or mutant (3′UTR-Mu) PICK1 3′-UTR along with miR-615-3p or control vector, anti-miR-615-3p or control oligo as indicated in MDA-MB-231 cells

To understand the mechanisms by which miR-615-3p promotes breast cancer cell migration, several computational methods were used to help identify miR-615-3p targets in humans. As miR-615-3p increased Smad2 and Smad3 phosphorylation, we hypothesized that its potential targets should decrease modulate TβRI stability. Among those potential targets, protein interacting with C-kinase-1(PICK1) is of particular interest because previously PICK1 was shown to antagonize transforming growth factor-beta (TGF-β) signaling by targeting TGF-β type I receptor (TβRI) for degradation and acted as an important negative regulator of TGF-β signaling [21, 32]. Therefore, we decided to carry out a series of experiments to determine whether PICK1 is a direct target of miR-615-3p. First, we detected the effect of miR-615-3p on PICK1 expression. Although miR-615-3p overexpression did not change PICK1 mRNA levels (Fig. 4a), there was a clear reduction in PICK1 protein levels in MCF-7 and T47D cells (Fig. 4b, upper panel). However, miR-615-3p silencing caused an increase in PICK1 protein levels in MDA-MB-468 and MDA-MB-231 cells (Fig. 4b, lower panel). These findings suggest that miR-615-3p targets PICK1 through translational inhibition. Next, we generated wild-type and mutant PICK1–3′-UTR expression plasmids that were fused to a luciferase reporter according to the matched sequence between PICK1–3′-UTR and miR-615-3p (Fig. 4c). We found miR-615-3p overexpression significantly inhibited luciferase activity of wild-type but not mutant reporter genes in MCF-7 cells (Fig. 4d, left). However, miR-615-3p silencing specifically enhanced the luciferase activity of wild-type but not mutant PICK1–3′-UTR (Fig. 4d, right).

### miR-615-3p overexpression and PICK1 inhibition produce similar changes, which are rescued by PICK1 ectopic expression in vitro

**Fig. 5:**
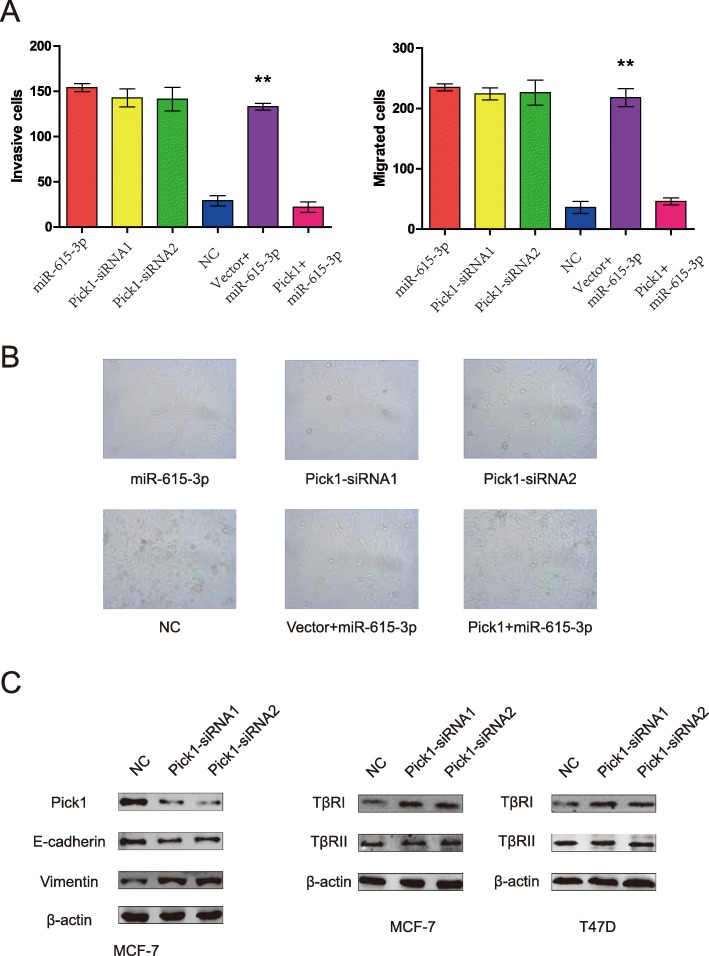
miR-615-3p overexpression and PICK1 inhibition produce similar changes, which are restored by PICK1 ectopic expression in vitro. (**a**) Transwell invasion and migration assays of MCF-7 cells were performed after transfection with negative control (NC), siRNA against PICK1, and/or miR-615-3p, PICK1(lacks the 3′UTR) as indicated. (**b**) Phase contrast microscopy of MCF-7 cells transfected as indicated (scale bars, 50 μm). (**c**) MCF-7 cells were transfected with PICK1 siRNAs, then PICK1, E-cadherin, Vimentin, TβRI and TβRII protein levels were detected by Western blot analysis (left panel). Western blot analysis was used to determine the effect of PICK1 knockdown on TβRI and TβRII expressions in MCF-7 and T47D cells (right two panels). β-actin served as an internal control

We further found that knockdown of PICK1 produced similar changes in invasion and migration assay to that of miR-615-3p overexpression (Fig. 5a). To determine whether these effects depend specifically on PICK1 suppression, we used an expression construct that encodes the entire PICK1 coding sequence but lacks the 3′UTR. Unsurprisingly, the re-expression of PICK1 completely inhibited miR-615-3p–mediated invasion and migration (Fig. 5a). This suggests that, after miR-615-3p overexpression, a decrease in PICK1 is required for cells to show increased invasiveness and migration.

Then we ask whether PICK1 connects miR-615-3p and EMT. The loss of E-cadherin is the hallmark of EMT. Interestingly, MCF-7 cells changed from round to a spindle-like mesenchymal phenotype after knockdown of PICK1 (Fig. 5b), which mimic the effect of miR-615-3p on EMT. Furthermore, knockdown of PICK1 also significantly reduced the E-cadherin but increased the Vimentin levels (Fig. 5c).

The TGF-β signaling network plays an essential role in the EMT of breast cancer cells. To determine whether PICK1 regulation of EMT is functionally associated with the TGFβ signaling pathway which is important for miR-615-3p-mediated EMT, we analyzed the expression levels of TβRI and TβRII in MCF7 and T47D cells with PICK1 knockdown. Among the TβRs, the amount of TβRI significantly increased in MCF7 and T47D cells on PICK1 knockdown, though little change was detected in TβRII (Fig. 5c). These results clearly show that PICK1 regulates TβRI expression in breast cancer cells.

Taken together, these data indicated that the ability of miR-615-3p to promote metastasis is attributable, in significant part, to its capacity to inhibit PICK1.

### High miR-615-3p and low PICK1 levels correlate with aggressive characteristics of clinical breast cancers

**Fig. 6:**
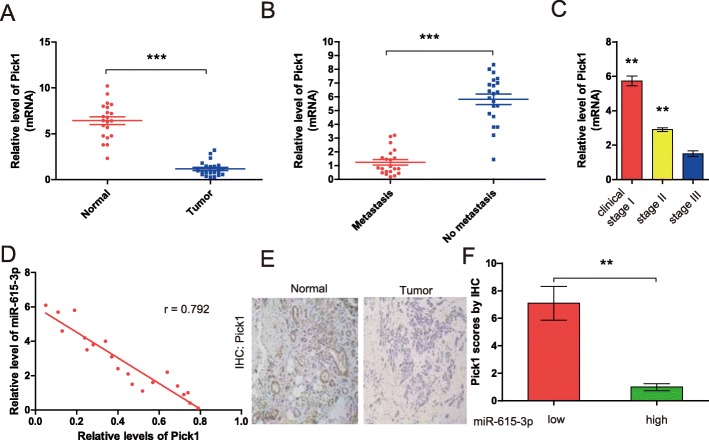
PICK1 expression in human breast cancer tissues and its correlation with miR-615-3p expression. (**a**) The qRT-PCR analysis of PICK1 levels in human breast cancer tissues and corresponding non-cancerous tissues (*n* = 21). (**b**) The qRT-PCR analysis of PICK1 mRNA levels in breast cancer tissues with (n = 21) or without (n = 21) lymphatic metastasis. (**c**) The qRT-PCR analysis of PICK1 mRNA levels in breast cancer patients at different clinical stages (WHO I, *n* = 11; WHO II, *n* = 20; and WHO III, n = 11). (**d**) The qRT-PCR analysis of PICK1 mRNA levels in breast cancer patients with low and high miR-615-3p levels. The relationship between PICK1 and miR-615-3p was analyzed by Pearson’s correlation. (**e**) Representative IHC images of PICK1 in normal and breast cancer tissues with various scores (100× magnifications). The staining scoring was obtained by intensity*the percentage of positive cells. The intensity of the IHC staining was graded as 0 (no staining), 1 (weak staining = light yellow), 2 (moderate staining = yellow brown) and 3 (strong staining = brown). The proportion of positively stained tumor cells in a field was scored as less than 5% scored 0, 5–20% scored 1, 20–50% scored 2, 50–75% scored 3, and > 75% scored 4. (**f**) IHC scores of the PICK1 protein level in breast cancer patients with low and high miR-615-3p levels

We next examined the clinical relevance of altered miR-615-3p and PICK1 expression in human breast cancers. We found that the levels of the PICK1 mRNA were lower in breast cancer tissues than in non-cancerous tissues (Fig. 6a) and correlated with lymphatic metastasis and the histological grade of the tumor (Fig. 6b and c). We also investigated the relationship between miR-615-3p expression and the mRNA and protein levels of PICK1 in breast cancer patients. The level of miR-615-3p was inversely correlated with those of the PICK1 mRNA (Fig. 6d). We measured PICK1 protein levels in human breast cancer and matched non-cancerous tissues using immunohistochemical staining (Fig. 6e). The level of miR-615-3p was inversely correlated with those of the PICK1 protein levels (Fig. 6f). suggesting that miR-615-3p promotes the malignant phenotypes of breast cancer by targeting PICK1.

### PICK1 inhibits the binding of DICER1 to Smad2/3 and the processing of pre-miR-615-3p to mature miR-615-3p

**Fig. 7:**
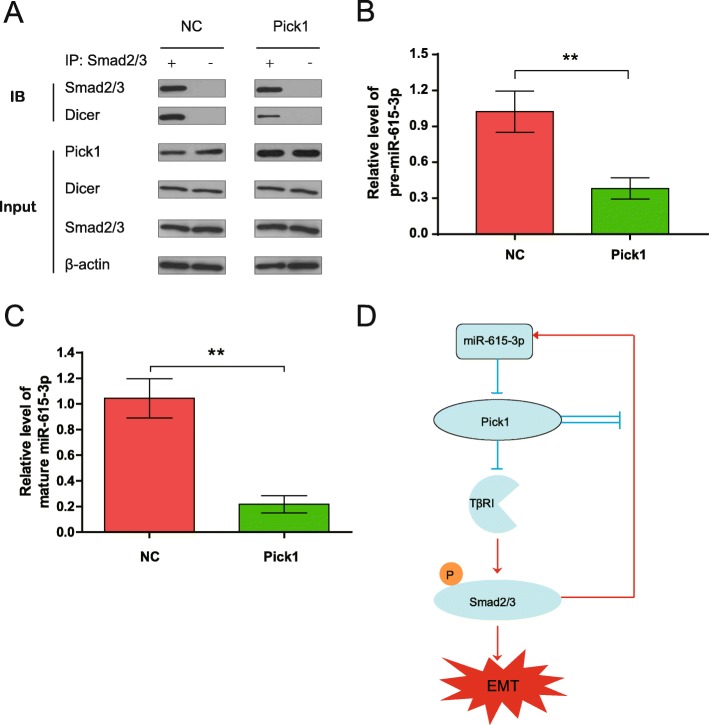
PICK1 disrupts the SMAD2/3-DICER1 complex and the processing of pre-miR-615-3p. **a** MDA-MB-231 cells were transfected with PICK1 or control vectors, and cell lysates were immunoprecipitated with a SMAD2/3 antibody (IP) or a rabbit IgG (control). Representative images show IP and control samples immunoblotted (IB) for PICK1, DICER1, and SMAD2/3. Input: representative western blot of input lysates showing DICER1 expression and β-actin as a loading control. **b** miRNAs were extracted from the IP and subjected to qRT-PCR assay to detect co-precipitation of pre-miR-615-3p with DICER1 and SMAD2/3. Pre-miR-615-3p expression was normalized and relativized to their correspondent controls. **c** qRT-PCR assay to detect mature miR-615-3p. **d** A schematic illustration of the positive feedback loop. miR-615-3p activates Smad2 and Smad3 signaling by suppressing PICK1. In turn, PICK1 downregulates miR-615-3p through disrupting the SMAD2/3-DICER1 complex and the processing of pre-miR-615-3p

Given that SMAD effectors of TGF-β1 signaling can interact with DICER1 to promote primary miR-21 processing into precursor miR-21 [33], We hypothesize that overexpression of PICK1 disrupts the association of p-SMAD2 and p-SMAD3 with DICER1 and thus decreases the processing of pre-miR-615-3p to mature miR-615-3p in breast cancer cells. To assess this hypothesis, we transfected MDA-MB-231 cells with PICK1 overexpression or control vectors, and then performed co-immunoprecipitation–RT-qPCR studies using an antibody that targets SMAD2/3. We found that DICER1 co-precipitated along with SMAD2/3 in the control group, but this binding was significantly decreased in MDA-MB-231 cells with PICK1 overexpression (Fig. 7a). After RNA bound to the protein complexes was isolated, a significant decrease of pre-miR-615-3p in MDA-MB-231 cells with PICK1 overexpression was observed (Fig. 7b). When the anti-SMAD2/3 antibody was substituted by a non-specific IgG in control assays, pre-miR-615-3p was absent of the immunoprecipitated complexes (data not shown). RT-qPCR studies showed a 5.1 fold decrease of mature miR-615-3p in MDA-MB-231 cells with PICK1 overexpression compared to control (Fig. 7c). Together, our data suggest that PICK1 inhibits the binding of DICER1 to Smad2/3 and the processing of pre-miR-615-3p to mature miR-615-3p in breast cancer cells, thus exerting a negative feedback loop (Fig. 7d).

## Discussion

Accumulating pieces of evidence have indicated that the miRNA system plays a critical role in the process of EMT [15, 34]. In our attempt to identify miRNAs expressed in breast cancer cells, we found that the amount of miR-615-3p is elevated in breast cancer cells and tissues compared with normal controls, ectopic miR-615-3p expression in breast cancer cells promoted EMT traits. The tumor-promoting role of miR-615-3p is linked to its ability to directly target 3′-UTR of PICK1. However, TβRI knockdown by siRNA or inhibition with SB431542 is sufficient to revert breast cancer cell migration and invasion enhanced by miR-615-3p. Opposite to miR-615-3p, PICK1 is preferentially expressed in human normal tissues and cells. The inverse correlation between miR-615-3p and PICK1 expression is consistent with our finding that miR-615-3p promotes EMT by diminishing PICK1 expression. In turn, PICK1 inhibits the binding of DICER1 to Smad2/3 and the processing of pre-miR-615-3p to mature miR-615-3p in breast cancer cells, thus exerting a negative feedback loop. These findings have implications for the potential application of miR-615-3p/PICK1/Smad2/3 axis in breast cancer treatment.

miRNAs have been implicated in almost all aspects of cancer biology, including angiogenesis, drug resistance, apoptosis, proliferation, invasion, and metastasis [35]. It has been shown that miRNAs may act as tumor suppressors or oncogenes in many cancers, depending on which pathways or genes they regulate [36]. However, very little was known about the role of miR-615-3p in tumor biology. A recent study has described that miR-615-3p is significantly upregulated in HCC patients with recurrence compared to the patients without recurrence [37]. Although in vitro experiments demonstrated that the miR-615-3p expression level is significantly correlated with malignant characteristics in HCC cell, the direct downstream genes targeted by miR-615-3p was not identified. Consistent with this report, our data indicate that miR-615-3p overexpression significantly promoted the in vitro migration and invasion, and in vivo pulmonary metastasis of breast cancer cells. Moreover, Subsequent mechanism studies revealed that miR-615-3p promoted EMT program by directly targeting PICK1. These findings along with previous studies indicate that miR-615-3p may mainly function as an onco-miRNA. In the future, there will be more targeted genes to be identified in other cancers.

Mechanistically, the increase of miR-615-3p in breast cancer may imply that miR-615-3p has an important role in signaling regulation. Indeed, we found that miR-615-3p specifically inhibits PICK1, leading to the stabilization of TGFBRI and the activation of the transcription factors SMAD2 and SMAD3. Although many miRNAs have been implicated in disrupting or increasing the TGF-β pathway [37]. To our knowledge, this is the first study to report that miRNA can regulate the PICK1 level. Indeed, PICK1 has an important role in breast cancer and other cancer initiation [19]. Thus, this study provides an alternative mechanism to regulate PICK1 for miRNAs.

Apparently, one miRNA may regulate many genes as its targets, while one gene may be targeted by many miRNAs [38]. Thus, miR-615-3p is very likely to regulate other genes simultaneously to promote breast tumor growth. In addition, PICK1 can also be targeted by other miRNAs than miR-615-3p. We postulated that one signal axis such as miR-615-3p/PICK1 has a critical role in specific cellular signaling and its function in breast cancer depends on the genetic context. Despite multiple relationships, our data provide solid evidence that the miR-615-3p/PICK1 axis has an important role in breast cancer cell migration and invasion. Thus, we strongly believe that targeting this pathway may be a potential therapeutic approach for the treatment of breast cancer.

The involvement of PICK1 in tumorigenesis has been suggested in several human cancers [21]. For example, PICK1 expression is down-regulated in grade IV astrocytic tumor cell lines and also in clinical cases of the disease in which grade IV tumors have progressed from lower-grade tumors. Exogenous expression of PICK1 in the grade IV astrocytic cell line U251 reduces their capacity for anchorage-independent growth, two-dimensional migration, and invasion through a three-dimensional matrix, strongly suggesting that low PICK1 expression plays an important role in astrocytic tumorigenesis [20]. However, we found that PICK1 is hardly detectable in breast cancer tissue but exhibits a higher level in normal breast tissue. The knockdown of PICK1 confers epithelial-like breast cancer cells with the ability to invade Matrigel and to disseminate. The consistency seen in the in vivo model and the excellent correlation between PICK1 expression and survival of patients with breast cancer in breast tumors supports the role of PICK1 in breast tumorigenicity established by our experimental studies. Based on this, one potential treatment option could be a combination of tamoxifen and a miR-615-3p inhibitor, which would work synergistically to prevent breast cancer tumorigenesis by controlling the process of EMT.

Herein, we assess the role of PICK1 on the TGF-β signaling effectors SMAD2/3 and DICER1 in the processing of pre-miR-615-3p to yield mature miR-615-3p in breast cancer cells. We also explored the functional coupling between activated SMAD2/3 and DICER1 to control the biogenesis of miR-615-3p in breast cancer. As expected, it was found that PICK1 controls the posttranscriptional processing of miR-615-3p through a direct protein-protein interaction between p-SMAD2/3 and the ribonuclease DICER1 in the pre-miR-615-3p maturation complex. This new TGF-β-dependent regulatory mechanism is consistent with previous reports that p-SMAD2/3 interacts with DICER1 or Drosha to promote pre-miR-21 processing [33]. Recently, PICK1 was reported to regulate miRNA-mediated translational repression by controlling Ago2 localization [39]. It will be interesting to demonstrate if Ago2 is implicated in miR-615-3p-mediated PICK1 repression in breast cancer cells.

## Conclusions

In summary, our data strongly suggest that concurrent gain of miR-615-3p and loss of PICK1 expression may be an important step in breast tumor progression and metastasis, highlighting the potential role of miR-615-3p in the prognostic evaluation and therapeutic application for breast cancers.

## Data Availability

All data generated or analyzed during this study are included in this article.

## Abbreviations

EMT: epithelial-mesenchymal transition
miRNAs: microRNAs
qRT-PCR: quantitative real-time polymerase chain reaction
TGF-β: The transforming growth factor-β
PICK1: Protein interacting with C kinase 1
ATCC: American type culture collection
3′-UTR: 3′-untranslated regions
